# Estimation of kidney doses from [^177^Lu]Lu-DOTA-TATE PRRT using single time point post-treatment SPECT/CT

**DOI:** 10.1186/s40658-024-00665-9

**Published:** 2024-07-25

**Authors:** Safia Spink, Daniel Gillett, Sarah Heard, Ines Harper, Ruth Casey, Luigi Aloj

**Affiliations:** 1 Department of Nuclear Medicine, Cambridge University Hospitals NHSFT, Cambridge, UK; 2 Department of Endocrinology, Cambridge University Hospitals NHSFT, Cambridge, UK; 3 Department of Genetics, University of Cambridge, Cambridge, UK; 4 Department of Radiology, University of Cambridge, Cambridge, UK

**Keywords:** Dota-tate, PRRT, Dosimetry, STP, Single time point, Lutate

## Abstract

**Background:**

Dosimetry after [^177^Lu]Lu-DOTA-TATE therapy can be demanding for both patients and the clinical service due to the need for imaging at several time points. In this work we compare three methods of single time point (STP) kidney dosimetry after [^177^Lu]Lu-DOTA-TATE therapy with a multiple time point (MTP) dosimetry method.

**Method:**

Method 1 (MTP): Kidney doses were calculated from 31 patients including 107 therapy cycles. Post-therapy SPECT images were acquired on day 0, 4 and 7 along with a CT scan on day 4. A mono-exponential fit was used to calculate kidney doses using cycle specific data. Method 2 (Consistent effective half-life): The effective half-life $\left({\text{T}}_{\text{e}\text{f}\text{f}}\right)$ calculated in cycle 1 was assumed consistent for subsequent cycles of therapy and the activity scaled using a single day 3–5 SPECT/CT. Methods 3 and 4 (Hänscheid and Madsen approximations): The Hänscheid approximation and Madsen approximation were both evaluated using a single SPECT/CT acquired on day 0, 4 and 7. All STP methods were compared to the MTP method for accuracy.

**Results:**

Using the MTP method, mean right and left kidney doses were calculated to be 2.9 *±* 1.1 Gy and 2.8 *±* 0.9 Gy respectively and the population ${\text{T}}_{\text{e}\text{f}\text{f}}$ was 56 *±* 13 h. For the consistent ${\text{T}}_{\text{e}\text{f}\text{f}}$, Hänscheid and Madsen methods, the percentage of results within *±* 20% of MTP method were 96% (*n* = 70), 95% (*n* = 80) and 94% (*n* = 80) respectively.

**Conclusion:**

All three single time point methods had > 94% of results within *±* 20% of the MTP method, however the consistent ${\text{T}}_{\text{e}\text{f}\text{f}}$ method resulted in the highest alignment with the MTP method and is the only method which allows for calculation of the patient-specific ${\text{T}}_{\text{e}\text{f}\text{f}}$. If only a single scan can be performed, day 4 is optimal for kidney dosimetry where the Hänscheid or Madsen approximation can be implemented with good accuracy.

## Introduction

PRRT with [^177^Lu]Lu-DOTA-TATE (Lutate) was first approved as a therapy by the European Medicines Agency in 2017 [1] and the Food and Drugs Administration in 2018 [2] based on the results of the randomised international NETTER-1 clinical trial [3]. Since then, it has been widely used as a treatment for metastatic somatostatin receptor positive neuroendocrine tumours (NETs) [4–7]. The treatment is administered over a standard course of 4 × 7.4 GBq intravenous infusions at 8-week intervals. A 23 Gy cumulative absorbed dose limit to the kidneys is adhered to with the standard course of treatment, which is considered safe for the kidneys and bone marrow - the organs at risk (OAR) [8, 9]. However, this dose limit is based on external beam radiotherapy in which the delivery of radiation differs from molecular radiotherapy [10].

Several studies have demonstrated that serious kidney toxicity rarely occurs with the current prescription of Lutate, with patients only experiencing a minor decrease in kidney function [7, 11–14]. Furthermore, studies have also shown that approximately 50% of patients could have received more cycles of therapy before reaching the 23 Gy dose limit [15] and in one study no severe renal toxicity was observed after cumulative administered activity as high as 78.6 GBq [16]. The calculation of doses delivered to target volumes and OAR for patients undergoing radionuclide therapy is best practice, but there are challenges to implement it routinely in clinical service. Post-therapy imaging is typically required at multiple time points to accurately sample the time activity curve (TAC), however, it is onerous for patients to make repeated visits to the hospital and requires significant clinical time and resources. To overcome this, several single time point (STP) methods of kidney dosimetry have been investigated to simplify the process.

The use of a patient-specific effective half-life $\left({\text{T}}_{\text{e}\text{f}\text{f}}\right)$ determined from multiple time point (MTP) imaging in the initial cycle of therapy with STP imaging in subsequent cycles has previously been investigated. This has been shown to be most accurate when using STP imaging at 24 h in subsequent cycles. Ardenfors et al. reported median doses in relation to true doses of 1.01 and a maximum difference in dose of -18% when using ${\text{T}}_{\text{e}\text{f}\text{f}}$ from cycle 1 and 24-hour imaging [17]. Willowson et al. reported average and maximum deviations of 2% and 45% when using the same method [18] and Sundlov et al. found differences of 1 ± 17% (2 standard deviations) [19].

Mathematical models such as physiologically based pharmacokinetic (PBPK) and non-linear mixed effects (NLME) have been used to predict kidney and tumour doses using STP measurements [20–22]. A popular model proposed by Hänscheid et al. requires a single SPECT/CT at 96 h after therapy to approximate the time-integrated activity (TIA) with the lowest maximum errors [23]. Hou et al. implemented this method and found that a scan time of 72 h was optimal [24]. This method has been widely accepted as a good approximation of TIA and is integrated into clinically approved dosimetry software [25]. Madsen et al. introduced a model that utilized simulations to estimate TIA from a single time sample based on the mean time of the rate constant, although prior knowledge of population averages of kinetic parameters is necessary [26]. This method has been implemented in several simplified dosimetry studies [17, 18, 27, 28]. More recently, Devasia et al. developed a NLME model using biodistribution data from SPECT/CT at 96 h to predict kidney TACs. The model accurately estimated kidney TIA with a median relative deviation of -3% and a reduced number of TACs with significant deviations compared to calculations using the entire data set [22]. The number of TACs with a relative deviation of > 10% was reduced by over 50% compared to Hänscheid [23] and Madsen [26] methods.

In this work, we share our centre’s own experience, taking into account the various practical considerations inherent in patient cohorts and scheduling constraints within a busy clinic. Our contribution adds to the expanding body of evidence supporting the use of STP dosimetry for Lutate therapy, specifically for estimating kidney doses. We compare three STP dosimetry methods to an MTP method to assess accuracy.

## Method

### Dataset

35 patients underwent a total of 122 cycles of Lutate therapy at Addenbrooke’s Hospital, Cambridge, UK between October 2018 and March 2022 with SPECT and SPECT/CT images acquired as part of their post-therapy imaging regime. 1 patient was excluded due to having only 1 cycle of therapy, 2 cycles were excluded due to a lack of post-therapy imaging, and 12 cycles were excluded due to complications in attenuation correction of the SPECT images from mismatched SPECT and CT images.

### Acquisition and reconstruction parameters

For logistical reasons and depending on camera availability, SPECT images were acquired using a GE Discovery NM 630 or NM/CT 670 and SPECT/CT images were acquired with the latter. Using Medium Energy General Purpose (MEGP) collimators, a SPECT acquisition was made using a 15% energy window centred upon the 208 keV photopeak. Scans were acquired feet first supine in H mode with body contour on. 30 steps were acquired per head, each covering an arc angle of 180° with a frame phase duration of 30 s using a 128 × 128 matrix and 4.41 mm pixel size. The CT image was acquired at 120 kV with Smart mA set to give a maximum of 120 mA and a noise index of 40. The slice thickness was 2.5 cm. The reconstruction parameters used for SPECT imaging included OSEM reconstruction with 2 iterations and 10 subsets, a configuration that was locally optimised for quantification and clinically acceptable noise suppression. Additionally, a Butterworth post-filtering technique was applied with a filter order of 0.3 and a cut-off value of 10. Scatter correction and resolution recovery were not applied in this case. The same acquisition and reconstruction parameters used for patient images were also used when performing phantom work to calculate a sensitivity factor for ^177^Lu on our gamma cameras.

### Kidney segmentation

**Fig. 1:**
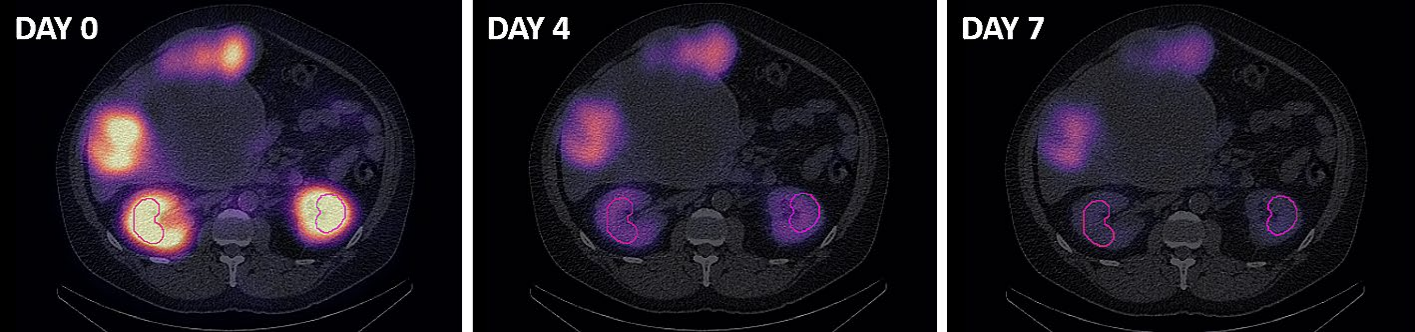
A small volume of interest drawn within the left and right kidneys on the day 0 SPECT and propagated onto the day 4 and 7 SPECT images

Segmentation of the kidneys was performed in 3D Slicer 4.11 [29]. A small volume of interest (VOI) at least 4 cm^3^ was placed in the cortex of normal kidney exhibiting homogenous distribution of radioactivity, as can be seen in Fig. 1. It has been shown that this method provides results less than 5–10% different from segmenting the entire organ [30–32]. A segment statistics quantification module in 3D Slicer computed the volume and number of voxels in the VOI which were used to quantify the activity.

### Method 1: multiple time point kidney dosimetry

Post-therapy SPECT imaging was performed on Day 0, 4 and 7. All were corrected for attenuation using a CT acquired on day 4. Images acquired on day 0 and 7 were reconstructed following co-registration of the day 4 CT using Volumetrix MI on GE Xeleris 4.0. There were occasional variations in the imaging schedule and 9 patients only had scans at two time points for all cycles of therapy. Kidney activity was quantified using the formula:

1 $$\text{Activity}\,\left(\text{MBq}\right) =\frac{\text{Standard}\,\text{kidney}\,\text{volume}\,\times\, \text{mean}\,\text{counts}\,\text{per}\, \text{voxel}}{\text{Voxel}\,\text{size}\, \times \text{system}\, \text{sensitivity}}$$

where the system sensitivity was 9297 cts/MBq and standard ICRP 110 adult male and adult female kidney volumes were used [29]. A TAC was made by fitting a monoexponential curve to the points using a least squares fitting, from which the y-intercept (${A}_{0}$) and decay coefficient ($\lambda$) were calculated. The TIA was calculated from integrating the TAC described by the equation:

2 $$\stackrel{\sim}{A} \left(\text{M}\text{B}\text{q}\cdot \text{s}\right)= {A}_{0} {\int }_{0}^{\infty }{e}^{-\lambda t} dt= \frac{{A}_{0}}{\lambda }$$

Standard ICRP 110 S-values were subsequently used to calculate absorbed kidney doses [33, 34].

### Method 2: using a patient-specific effective half-life

The patient-specific effective half-life was calculated from post-therapy imaging in cycle 1 using a minimum of two images. The first image was acquired within 24 h of therapy and the second image was acquired no earlier than 96 h post therapy. The ${\text{T}}_{\text{e}\text{f}\text{f}}$ was assumed to be consistent for all cycles of therapy. In subsequent cycles a single SPECT/CT acquired on day 3–5 was used to scale the TAC for activity concentration. Decay correction of the kidney activity was performed using the cycle 1 ${\text{T}}_{\text{e}\text{f}\text{f}}$ to estimate kidney doses. Dose estimates in subsequent cycles using this method were compared to calculations using the MTP method which used the full set of data acquired in cycles 2–4.

### Method 3 and method 4: Hänscheid and Madsen approximations

A single SPECT/CT after each cycle of therapy was used to estimate the TIA using both the Hänscheid approximation and the Madsen approximation with a population effective half-life$\left({\text{T}}_{\text{p}-\text{e}\text{f}\text{f}}\right)$. The Hänscheid approximation is described by the equation:

3 $$\stackrel{\sim}{\left(A\right)}=\frac{1}{\text{l}\text{n}\left(2\right)}\ A\left({T}_{SC}\right)\ 2{T}_{SC}$$

Where $A\left({T}_{SC}\right)$ is the activity in a VOI at scan time${T}_{SC}$, and the Madsen approximation described by the equation:

4 $$\stackrel{\sim}{\left(A\right)}=\frac{1}{\text{l}\text{n}\left(2\right)}\ A\left({T}_{SC}\right)\ {2}^{\frac{{T}_{sc}}{{T}_{p-eff}}}\ {T}_{p-eff}$$

The${\text{T}}_{\text{p}-\text{e}\text{f}\text{f}}$was calculated as an average of the right and left kidney from all 107 cycles of therapy. The accuracy of these approximations in comparison to the MTP method was investigated using a single scan acquired on day 0, 4 and 7, and the proportion of results within *±* 10% and 20% of the MTP method was calculated to consider which time point is most suitable for clinical practice.

## Results

### MTP method

107 cycles of therapy from 31 patients were analysed using the MTP method. The mean and standard deviation of the right and left kidney doses from each cycle were calculated to be 2.9 *±* 1.1 Gy and 2.8 *±* 0.9 Gy, respectively. No patients exceeded the 23 Gy cumulative dose limit.

### Consistent effective half-life method

**Fig. 2:**
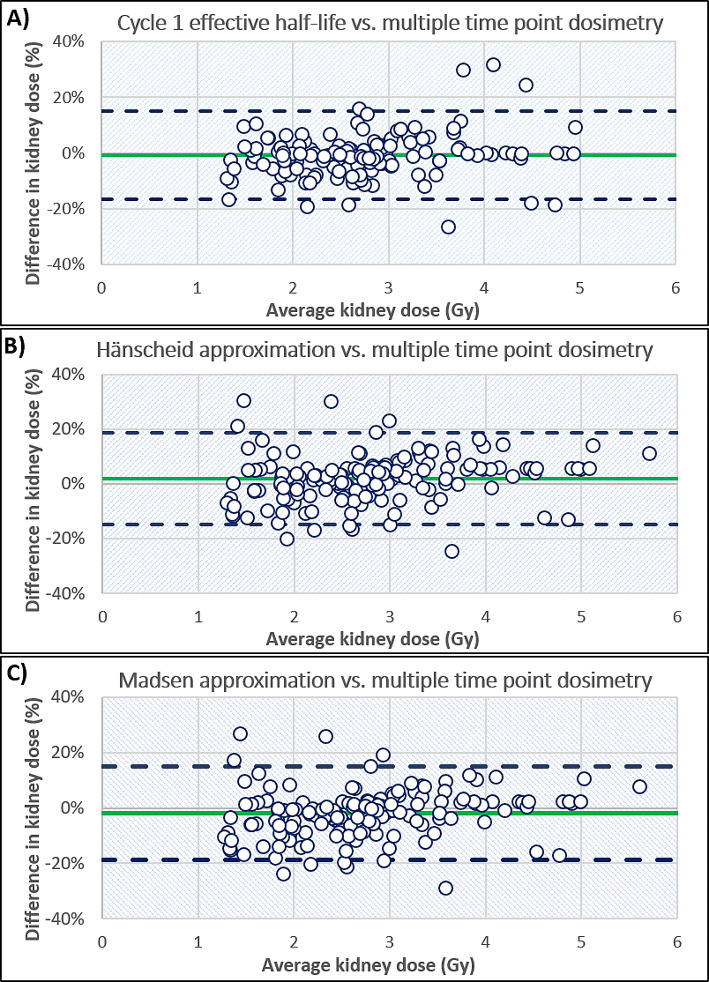
Bland-Altman plots assessing the agreement between the multiple time point dosimetry method and **A**) consistent effective half-life method **B**) Hanscheid approximation **C**) Madsen approximation for kidney dose calculations. The x-axis displays average kidney doses and the y-axis shows the percentage difference relative to the multiple time point method. The green solid line indicates the mean difference between the two methods and the blue dashed lines indicate the mean *±* 1.96 standard deviations

Kidney doses were estimated for 70 subsequent cycles using a cycle 1 ${\text{T}}_{\text{e}\text{f}\text{f}}$. Compared to the MTP method, both kidneys exhibited a median difference of -0.4%. The right kidney showed a standard deviation $\left(\sigma \right)$ of 8.8% and an interquartile range (IQR) of 8.0%, while the left kidney exhibited a standard deviation of 7.3% and an IQR of 6.4%. When scaling the TAC using a single day 3, 4 and 5 image, the median difference in estimated dose in relation to the MTP method was 0% (*n* = 12), 0% (*n* = 53) and − 4% (*n* = 5) respectively. Using this method, 73% of results for both kidneys were within *±* 10% of the MTP method, and 96% of results were within *±* 20% of the MTP method. These results can be visualised in the Bland-Altman plot in Fig. 2A and in Table 1.

### Hänscheid method

**Fig. 3:**
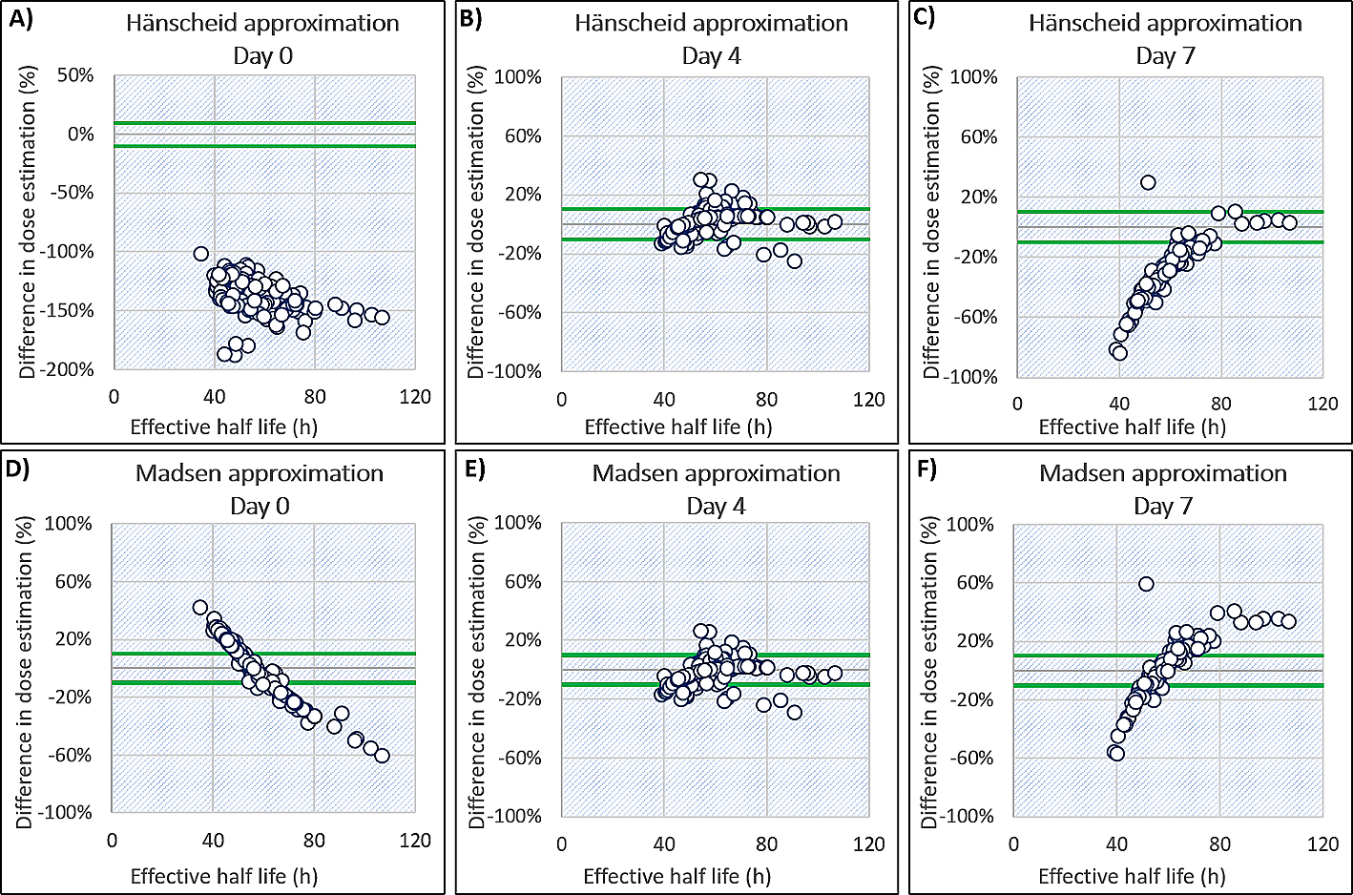
A comparison of the use of a single image on day 0, 4 and 7 to estimate kidney doses using the Hänscheid and Madsen approximations. Estimations are compared to doses calculated using MTP dosimetry. Green lines indicate a *±* 10% difference in relation to MTP dosimetry

When using the Hänscheid approximation for kidney dosimetry with a single SPECT scan acquired on day 0, 4 and 7, the mean difference in dose compared to the MTP method was − 137%, 2% and − 28% respectively. The percentage of results for which both kidney doses deviated less than *±* 10% from the MTP method was 0% (*n* = 81), 63% (*n* = 80) and 9% (*n* = 58) for STP imaging on day 0, 4 and 7, respectively. These results can be seen in Fig. 3A, B and C. When using a day 4 SPECT/CT, 95% of results were within *±* 20% of the MTP method. The comparison of the Hänscheid approximation and MTP method can be seen in the Bland-Altman plot in Fig. 2B and in Table 1.

### Madsen method

The mean and standard deviation of the population effective half-life were 56 *±* 13 h which was used for the Madsen approximation. For STP imaging on day 0, 4 and 7 a mean difference in time-integrated activity was seen of 0%, -2% and 2% respectively. The percentage of results for which both kidney doses deviated less than *±* 10% from the MTP method was 25% (*n* = 81), 66% (*n* = 80), and 28% (*n* = 58) for STP imaging on day 0, 4 and 7, respectively. These results can be seen in Fig. 3D, E and F. When using a day 4 SPECT/CT, 94% of results were within *±* 20% of the MTP method. The comparison of the Madsen approximation and MTP method can be seen in the Bland Altman plot in Fig. 2C; Table 1.

**Table 1:** Comparison of simplified methods of kidney dosimetry

Kidney dosimetry method	Mean dose (Gy)	Number of cycles	Results within 10% of MTP (%)	Results within 20% of MTP (%)	Max absolute difference (%)	Bland-Altman Mean (%)	Bland Altman 1.96$\varvec{\sigma }$ (%)
MTP	2.9	107	-	-	-	-	-
Consistent ${\text{T}}_{\text{e}\text{f}\text{f}}$	2.7	70	73	96	32	-1	16
Hänscheid (Day 0)	0.54	81	0	0	-188	-137	28
Hänscheid (Day 4)	2.8	80	63	95	31	2	17
Hänscheid (Day 7)	2.3	58	9	26	-83	-28	37
Madsen (Day 0) = 56 h	2.8	81	25	58	-77	0	39
Madsen (Day 4) = 56 h	2.7	80	66	94	27	-2	17
Madsen (Day 7) = 56 h	3.1	58	28	60	60	2	39

## Discussion

In Table 2, we compiled similar research findings, which were gathered through a PubMed database search using relevant keywords. These studies encompass all clinical research on kidney dosimetry for ^177^Lu-DOTATATE where STP methods were compared to a MTP reference method using SPECT and SPECT/CT imaging. Previous studies [17, 18, 35] have explored the utilisation of a patient-specific ${\text{T}}_{\text{e}\text{f}\text{f}}$, determined during the initial therapy cycle with STP imaging in subsequent cycles. Ardenfors et al. found that imaging on day 1 was optimal for kidney dosimetry compared to day 7. Willowson et al. demonstrated that STP imaging at 24 h in subsequent cycles was feasible, and more accurate than imaging at 4 h. Del Prete et al. utilised two time point imaging in cycle 1 to calculate the ${\text{T}}_{\text{e}\text{f}\text{f}}$, showing that STP imaging on day 3 was more accurate than at 24 h. Figure 2A illustrates that using the cycle 1 ${\text{T}}_{\text{e}\text{f}\text{f}}$ to estimate kidney doses is a particularly good approximation, with 96% of results deviating less than 20% from the MTP method and a maximum deviation of 32%. This method is most ideal when imaging occurs at three time points to calculate most accurately the ${\text{T}}_{\text{e}\text{f}\text{f}}$, however the ${\text{T}}_{\text{e}\text{f}\text{f}}$ was calculated using only two time points for 17 therapy cycles, with all results deviating less than 10% from the MTP method. Additionally, there were negligible differences in scaling the TAC using a day 3 or 4 scan. This method has the advantage over the Hänscheid and Madsen approximation methods because the patient-specific ${\text{T}}_{\text{e}\text{f}\text{f}}$ can be determined and be used to calculate personalised contact restrictions post-therapy.

The calculations by Hänscheid et al. suggested that a single SPECT/CT for kidney dosimetry is adequate at 72 h. However, if dosimetry is also to be performed on tissues with a longer ${\text{T}}_{\text{e}\text{f}\text{f}}$, a scan at 96 h is more appropriate. As depicted in Fig. 3B, we have demonstrated that the Hänscheid approximation is most optimal on day 4 compared to day 0 and day 7, with a maximum deviation from MTP dosimetry of 31%.

Variations in our imaging schedule meant that for 12 cycles of therapy, patients underwent a SPECT/CT on day 3 post-injection instead of day 4. This small dataset indicated that kidney dose estimates were within 10% of MTP dosimetry for 83% of cycles, with a maximum deviation of 18%. This finding suggests that imaging on day 3 might be more optimal than day 4 for kidney dosimetry. However, considering the literature, it is likely to be too early if tumour dosimetry is also being performed, due to the slower clearance. Overall, we showed good correlation between the Hänscheid approximation using day 4 imaging and MTP dosimetry as can be seen in Fig. 2B, however fewer results were within *±* 10% of the MTP method compared to use of a cycle 1 ${\text{T}}_{\text{e}\text{f}\text{f}}$.

Our results align well with other studies that have implemented the Hänscheid approximation [22, 24, 27, 28]. Peterson et al. demonstrated that a SPECT/CT at 71–126 h was optimal for both kidneys and tumours with maximum deviations of 26%. Devasia et al. found that imaging on day 4 yielded maximum deviations of -36%, with 80% of results showing less than a 10% difference from their standard dosimetry method. Hou et al. concluded that 72 h was optimal for kidney dosimetry [24].

The mean effective half-life of the kidneys for our population was determined to be 56 *±* 13 h, which aligns well with findings from previous studies by Hänscheid et al. [23]. (51 h), Zhao et al. [27]. (45 h), Willowson et al. [18]. (53 h), Garske et al. [36]. (52.6 h) and Sundlov et al. (51.6 h) [19]. The Madsen approximation was determined to be most effective when applied at a time point corresponding to the mean ${\text{T}}_{\text{p}-\text{e}\text{f}\text{f}}$ [26]. Subsequent studies have implemented this method with imaging conducted between days 3–5, resulting in mean deviations of 4% [28]. Additionally, in implementations with ${\text{T}}_{\text{p}-\text{e}\text{f}\text{f}}$ values of 52 h, imaging on day 1 resulted in all 77 results within *±* 20% of their standard method [17] while imaging on day 4 resulted in a mean bias of 0.7% [22]. Furthermore, day 3 imaging with a 45 h ${\text{T}}_{\text{p}-\text{e}\text{f}\text{f}}$[27] showed success with 98% of studies deviating less than 10% from the reference method, however a 54.2 h ${\text{T}}_{\text{p}-\text{e}\text{f}\text{f}}$ with 4 h and 24 h imaging showed large overestimates in kidney doses of 59% and 30% respectively [18]. From our research, the Madsen approximation using a ${\text{T}}_{\text{p}-\text{e}\text{f}\text{f}}$ of 56 h was shown to be slightly more accurate than the Hänscheid approximation when using a day 4 scan. However, this would require significant work to calculate a local ${\text{T}}_{\text{p}-\text{e}\text{f}\text{f}}$ and so using the Hänscheid approximation which is integrated into clinically approved dosimetry software would be more appropriate for routine dosimetry if only day 4 images are available.

In a similar approach to Devasia et al. [22] we assessed the percentage of results differing by 5%, 10%, 20% and 30% from MTP dosimetry. However, we limited the documented results to 10% and 20% because 5% was too stringent and 30% was not narrow enough to demonstrate differences.

Considering the conservative kidney toxicity limit of 23 Gy and the typical kidney doses reported in literature, which usually range around 4 Gy per cycle [35, 37], the total treatment dose could amount to 16 Gy. Hence, with a 20% margin of error, the potential error could reach 3.2 Gy. Given this calculation, similar to many others in the field, we consider this uncertainty to be sufficiently small for routine clinical practice. Crucially, this margin of error not only streamlines processes for both patients and the department but also provides ample sensitivity to detect doses that deviate significantly from the expected values and that might be clinically important and need to be dealt with accordingly.

**Table 2:** Comparison of our work with the relevant literature

Reference	Reference imaging schedule	STP method	Error/ Uncertainty compared to reference method	Patients
A. Chicheportiche [38]	SPECT/CT on day 1, 4 and 7	STP multiple-linear-regression model from imaging on day 7 in cycle 1 and day 1 in cycles 2–4	Relative difference of 0.8% ± 8.0%	159
C. Wang [39]	SPECT/CT on days 0, 1–2, 3–5 and 6–8	2 x STP expressions influenced by Madsen et al. and Hänscheid et al., with day 3–5 imaging	Mean absolute error < 7% (SD < 10%) for both STP methods	27
A.B. Peterson [28]	SPECT/CT day 0, 1 4 and 7	Hänscheid method (day 3–5 imaging)	Mean % error (S.D) − 3.1 (8.4)	28
		Madsen method (day 3–5 imaging)	Mean % error (S.D) -4.1 (7.4)	
O. Ardenfors [17]	SPECT/CT on day 1 and 7	Madsen method (day 1 imaging) T_eff_ = 52 h	Median ratio of doses in relation to reference method 1.00 (IQR: 0.97–1.03)	39
		Patient-specific T_eff_ determined for the initial cycle and SPECT/CT on day 1 of subsequent cycles	1.01 (IQR: 0.98–1.04)	
T. P. Devasia [22]	SPECT/CT up to 4 times between days 0 and 7	Nonlinear mixed model using day 4 imaging	Mean % bias 1.9 [− 19.2, 10.7]	250 virtual patients generated from 10 clinical patients
		Madsen method (day 4 imaging) T_eff_ = 52 h	0.7 [− 35.1, 17.5]	
		Hänscheid method (day 4 imaging)	2.7 [− 36.1, 18.5]	
W. Zhao [27]	SPECT/CT on day 0, 1 and 3	Madsen method (day 3 imaging) T_eff_ =45 h	Kidney absorbed dose deviated < 10% for 98% of studies	39
K.P. Willowson [18]	SPECT/CT on day 0, 1 and 4–5	Madsen method (4 h imaging and 24 h imaging) T_eff_ = 54.2 h	Average difference of 59% and 30% from MTP imaging when using 4 h data and 24 h data.	18
		Patient-specific half-life determined from C1 with STP imaging at 4 h and 24 h imaging	Average difference of 13% and 2% when using 4 h data only and 24 h data only.	
M. Del Prete [35]	SPECT/CT on day 0, 1 and 3	Hänscheid method with day 3 imaging	5.8% median relative error	79
		2 time point in cycle 1(day 1 + day 3) + day 1 STP hybrid method	3.4% relative error	
		2 time point in cycle 1 (day 1, day 3) + day 3 STP hybrid method	2.2% relative error	
Our work	SPECT on day 0 and 7, SPECT/CT on day 4	Hänscheid method (day 0, 4 and 7 imaging)	Mean difference of -137%, 2% and − 28% respectively	31
		Madsen method (day 0, 4 and 7 imaging) T_eff_ = 56 h	Mean difference of 0%, -2% and 2% respectively	
		Patient-specific T_eff_ calculated in cycle 1 scaled with a day 3–5 SPECT/CT	Mean difference of -1%	

## Limitations

MIRD pamphlets No. 23 and No. 26 present comprehensive guidelines and recommendations for quantitative SPECT imaging [40], and for imaging with ^177^Lu [41]. Scatter correction methods such as a triple energy window can enhance quantitative accuracy [42], however our research did not implement scatter correction. Additionally, for kidney image reconstruction using OSEM, MIRD recommends 40 updates to recover 90% of the maximum activity, whereas our protocol uses 20 updates. While our study primarily investigated various STP methods’ relative effects, our imaging protocol diverged from MIRD guidelines.

Molecular radiotherapy incorporates large uncertainties of 25–30% within the overall calculation of absorbed dose, with the error in the time-integrated activity the most significant contribution [43]. In our study, we encountered a challenge with manual rigid registration when attempting to align day 0 and day 7 SPECT images with a day 4 CT scan. This alignment proved impossible for 12 therapy cycles due to variations in patient positioning or the absence of distinct features to aid in alignment in each plane. This presented a potential issue since using these misaligned images would have led to gross inaccuracies in both the attenuation corrections to the SPECT image and the quantification of activity in the kidneys. To address this concern, we recommend conducting SPECT/CT acquisitions at each time point, despite the additional radiation dose involved, which can help to ensure more accurate dosimetry calculations. Furthermore, we used a small VOI method for segmentation of kidneys. The primary reason for this was to overcome the challenges associated with the large VOI method. Delineating the kidneys whilst avoiding the spill in effects from neighbouring organs, particularly those impacted by tumours proved difficult. Whilst acknowledging the potential limitation of the small VOI method in representing the concentration of the entire organ, it has been shown by Sandstrom et al. to be a viable method in achieving kidney dose estimates [32].

## Conclusion

Kidney dosimetry after Lutate therapy is necessary, although difficult to implement routinely due to the time and resources required. Single time point dosimetry methods can streamline the process for both patients and the department. Of the three STP methods investigated, using a cycle 1 ${\text{T}}_{\text{e}\text{f}\text{f}}$ and a single day 4 SPECT/CT in subsequent cycles gave the highest proportion of results in good alignment with the MTP method. If it is only possible for a patient to have a single scan after therapy, this should be a SPECT/CT performed on day 4 which allows the kidney doses to be approximated using either the Hänscheid or Madsen approximation which can both be used to estimate kidney doses with similarly accuracy, although the Hänscheid approximation is more accessible when the local ${\text{T}}_{\text{p}-\text{e}\text{f}\text{f}}$ is unknown.

## Data Availability

The datasets used and/or analysed during the current study are available from the corresponding author on reasonable request.

## Abbreviations

PRRT: Peptide receptor radionuclide therapy
NETs: Neuroendocrine tumours
OAR: Organs at risk
TAC: Time-activity curve
STP: Single time point
T_eff_: Effective half-life
MTP: Multiple time point
PBPK: Physiologically based pharmacokinetic
NLME: Non-linear mixed effects
TIA: Time-integrated activity
MEGP: Medium Energy General Purpose
OSEM: Ordered subset expectation maximization
VOI: Volume of interest
T_p-eff_: Population effective half-life

## References

[1] Authorization details for Lutathera^®^ in Europe. Accessed: Feb. 19, 2024. [Online]. Available: https://www.ema.europa.eu/en/medicines/human/EPAR/lutathera#authorisation-details-section.

[2] FDA Letter of Approval for LUTATHERA^®^. Accessed: Feb. 19, 2024. [Online]. Available: https://www.accessdata.fda.gov/drugsatfda_docs/appletter/2018/208700Orig1s000ltr.pdf.

[3] Strosberg J, et al. Phase 3 trial of 177 Lu-Dotatate for Midgut neuroendocrine tumors. N Engl J Med. Jan. 2017;376(2):125–35. 10.1056/NEJMOA1607427/SUPPL_FILE 10.1056/NEJMoa1607427 PMC5895095 28076709

[4] Bodei L et al. Sep., Peptide receptor radionuclide therapy with 177Lu-DOTATATE: the IEO phase I-II study, *European Journal of Nuclear Medicine and Molecular Imaging 2011 38:12*, vol. 38, no. 12, pp. 2125–2135, 2011, 10.1007/S00259-011-1902-1. 10.1007/s00259-011-1902-1 21892623

[5] Kwekkeboom DJ, et al. Treatment with the radiolabeled somatostatin analog [177 Lu-DOTA 0,Tyr3]octreotate: toxicity, efficacy, and survival. J Clin Oncol. 2008;26(13):2124–30. 10.1200/JCO.2007.15.2553. 18445841 10.1200/JCO.2007.15.2553

[6] Kwekkeboom DJ, et al. Radiolabeled somatostatin analog [177Lu-DOTA0,Tyr3]octreotate in patients with endocrine gastroenteropancreatic tumors. J Clin Oncol. Apr. 2005;23(12):2754–62. 10.1200/JCO.2005.08.066. 10.1200/JCO.2005.08.066 15837990

[7] Garske-Román U et al. Jun., Prospective observational study of 177Lu-DOTA-octreotate therapy in 200 patients with advanced metastasized neuroendocrine tumours (NETs): feasibility and impact of a dosimetry-guided study protocol on outcome and toxicity, *Eur J Nucl Med Mol Imaging*, vol. 45, no. 6, pp. 970–988, 2018, 10.1007/S00259-018-3945-Z. 10.1007/s00259-018-3945-z PMC5915504 29497803

[8] Erbas B, Tuncel M. Renal function Assessment during peptide receptor Radionuclide Therapy. Semin Nucl Med. Sep. 2016;46(5):462–78. 10.1053/J.SEMNUCLMED.2016.04.006. 10.1053/j.semnuclmed.2016.04.006 27553471

[9] Park EA, Graves SA, Menda Y. The Impact of Radiopharmaceutical Therapy on Renal Function, *Semin Nucl Med*, vol. 52, no. 4, pp. 467–474, Jul. 2022, 10.1053/J.SEMNUCLMED.2022.02.004. 10.1053/j.semnuclmed.2022.02.004 35314056

[10] Emami B, et al. Tolerance of normal tissue to therapeutic irradiation. Int J Radiat Oncol Biol Phys. May 1991;21(1):109–22. 10.1016/0360-3016(91)90171-Y. 10.1016/0360-3016(91)90171-y 2032882

[11] Bergsma H, et al. Nephrotoxicity after PRRT with (177)Lu-DOTA-octreotate. Eur J Nucl Med Mol Imaging. Sep. 2016;43(10):1802–11. 10.1007/S00259-016-3382-9. 10.1007/s00259-016-3382-9 PMC4969358 27160225

[12] Sundlöv A, et al. Individualised 177Lu-DOTATATE treatment of neuroendocrine tumours based on kidney dosimetry. Eur J Nucl Med Mol Imaging. Aug. 2017;44(9):1480–9. 10.1007/S00259-017-3678-4. 10.1007/s00259-017-3678-4 PMC5506097 28331954

[13] Valkema R et al. Long-term follow-up of renal function after peptide receptor radiation therapy with (90)Y-DOTA(0),Tyr(3)-octreotide and (177)Lu-DOTA(0), Tyr(3)-octreotate. J Nucl Med, 2005. 15653656

[14] Alsadik S et al. Feb., Safety of peptide receptor radionuclide therapy with 177Lutetium DOTATATE in neuroendocrine tumour patients with chronic kidney disease, *Journal of Nuclear Medicine*, vol. 63, no. 10, pp. 1503–1508, 2022, 10.2967/JNUMED.121.263056. 10.2967/jnumed.121.263056 PMC9536708 35210299

[15] Sandström M, et al. Individualized dosimetry of kidney and bone marrow in patients undergoing 177Lu-DOTA-octreotate treatment. J Nucl Med. Jan. 2013;54(1):33–41. 10.2967/JNUMED.112.107524. 10.2967/jnumed.112.107524 23223392

[16] Del Prete M, Buteau F-A, Beaulieu A, Beauregard J-M. Personalized 177Lu-octreotate peptide receptor radionuclide therapy of neuroendocrine tumors: initial dosimetry and safety results of the P-PRRT trial, *Journal of Nuclear Medicine*, vol. 58, no. supplement 1, pp. 242–242, May 2017, Accessed: Jun. 20, 2023. [Online]. Available: https://jnm.snmjournals.org/content/58/supplement_1/242.

[17] Ardenfors O, Nilsson JN, Thor D, Hindorf C. Simplified dosimetry for kidneys and tumors in 177Lu-labeled peptide receptor radionuclide therapy. EJNMMI Phys. Dec. 2022;9(1). 10.1186/s40658-022-00473-z. 10.1186/s40658-022-00473-z PMC9209556 35723797

[18] Willowson KP, Eslick E, Ryu H, Poon A, Bernard EJ, Bailey DL. Feasibility and accuracy of single time point imaging for renal dosimetry following 177 Lu-DOTATATE (‘Lutate’) therapy. EJNMMI Phys. Dec. 2018;5(1):1–9. 10.1186/S40658-018-0232-9/FIGURES/1. 10.1186/s40658-018-0232-9 PMC6300448 30569328

[19] Sundlöv A, et al. Feasibility of simplifying renal dosimetry in 177Lu peptide receptor radionuclide therapy. EJNMMI Phys. Dec. 2018;5(1). 10.1186/S40658-018-0210-2. 10.1186/s40658-018-0210-2 PMC6031553 29974391

[20] Hardiansyah D, Riana A, Beer AJ, Glatting G. Single-time-point estimation of absorbed doses in PRRT using a non-linear mixed-effects model. Z Med Phys. Feb. 2023;33(1). 10.1016/J.ZEMEDI.2022.06.004. 10.1016/j.zemedi.2022.06.004 PMC10082376 35961809

[21] Chicheportiche A, et al. Simple model for estimation of absorbed dose by organs and tumors after PRRT from a single SPECT/CT study. EJNMMI Phys. Dec. 2021;8(1):1–16. 10.1186/S40658-021-00409-Z/TABLES/4. 10.1186/s40658-021-00409-z PMC8390741 34436698

[22] Devasia TP, Dewaraja YK, Frey KA, Wong KK, Schipper MJ. A Novel Time-Activity Information-Sharing Approach Using Nonlinear Mixed Models for Patient-Specific Dosimetry with Reduced Imaging Time Points: Application in SPECT/CT After 177Lu-DOTATATE, *J Nucl Med*, vol. 62, no. 8, pp. 1118–1125, Aug. 2021, 10.2967/JNUMED.120.256255. 10.2967/jnumed.120.256255 PMC8833869 33443063

[23] Hänscheid H, Lapa C, Buck AK, Lassmann M, Werner RA. Dose mapping after endoradiotherapy with 177Lu-DOTATATE/DOTATOC by a single measurement after 4 days. J Nucl Med. Jan. 2018;59(1):75–81. 10.2967/JNUMED.117.193706. 10.2967/jnumed.117.193706 28588150

[24] Hou X, et al. Feasibility of single-time-point dosimetry for Radiopharmaceutical therapies. J Nucl Med. Jul. 2021;62(7):1006–11. 10.2967/JNUMED.120.254656. 10.2967/jnumed.120.254656 PMC8882881 33127625

[25] Capala J et al. Dec., Dosimetry for Radiopharmaceutical Therapy: Current Practices and Commercial Resources, *J Nucl Med*, vol. 62, no. Suppl 3, pp. 3S-11S, 2021, 10.2967/JNUMED.121.262749. 10.2967/jnumed.121.262749 PMC12079727 34857621

[26] Madsen MT, Menda Y, O’Dorisio TM, O’Dorisio MS. Technical note: single time point dose estimate for exponential clearance. Med Phys. May 2018;45(5):2318–24. 10.1002/MP.12886. 10.1002/mp.12886 PMC5948162 29577338

[27] Zhao W, Esquinas PL, Frezza A, Hou X, Beauregard JM, Celler A. Accuracy of kidney dosimetry performed using simplified time activity curve modelling methods: a 177Lu-DOTATATE patient study. Phys Med Biol. Aug. 2019;64(17). 10.1088/1361-6560/AB3039. 10.1088/1361-6560/ab3039 31287093

[28] Peterson AB, Mirando DM, Dewaraja YK. Accuracy and uncertainty analysis of reduced time point imaging effect on time-integrated activity for 177Lu-DOTATATE PRRT in patients and clinically realistic simulations. EJNMMI Res. Jun. 2023;13(1):1–13. 10.1186/S13550-023-01007-Z/TABLES/3. 10.1186/s13550-023-01007-z PMC10260735 37306783

[29] Fedorov A, et al. 3D slicer as an image computing platform for the quantitative Imaging Network. Magn Reson Imaging. Nov. 2012;30(9):1323–41. 10.1016/J.MRI.2012.05.001. 10.1016/j.mri.2012.05.001 PMC3466397 22770690

[30] Del Prete M, Buteau FA, Beauregard JM. Personalized 177Lu-octreotate peptide receptor radionuclide therapy of neuroendocrine tumours: a simulation study. Eur J Nucl Med Mol Imaging. Aug. 2017;44(9):1490–500. 10.1007/S00259-017-3688-2. 10.1007/s00259-017-3688-2 28361189

[31] Sandström M, Garske U, Granberg D, Sundin A, Lundqvist H. Individualized dosimetry in patients undergoing therapy with 177Lu-DOTA-D-Phe1-Tyr3-octreotate, *Eur J Nucl Med Mol Imaging*, vol. 37, no. 2, pp. 212–225, Feb. 2010, 10.1007/S00259-009-1216-8. 10.1007/s00259-009-1216-8 19727718

[32] Sandström M, Ilan E, Karlberg A, Johansson S, Freedman N, Garske-Román U. Method dependence, observer variability and kidney volumes in radiation dosimetry of 177Lu-DOTATATE therapy in patients with neuroendocrine tumours, *EJNMMI Phys*, vol. 2, no. 1, pp. 1–13, Dec. 2015, 10.1186/S40658-015-0127-Y. 10.1186/s40658-015-0127-y PMC4883125 26501825

[33] ICRP. Adult reference computational phantoms. ICRP Publication 110., 2009. 10.1016/j.icrp.2009.09.001 19897132

[34] Chauvin M et al. Oct., OpenDose: Open-Access Resource for Nuclear Medicine Dosimetry, *J Nucl Med*, vol. 61, no. 10, pp. 1514–1519, 2020, 10.2967/JNUMED.119.240366. 10.2967/jnumed.119.240366 PMC7539649 32169912

[35] Prete MD, et al. Accuracy and reproducibility of simplified QSPECT dosimetry for personalized 177Lu-octreotate PRRT. EJNMMI Phys. Dec. 2018;5(1):1–20. 10.1186/S40658-018-0224-9/FIGURES/6. 10.1186/s40658-018-0224-9 PMC6186532 30318563

[36] Garske U, et al. Minor changes in effective half-life during fractionated 177Lu-octreotate therapy. Acta Oncol. Jan. 2012;51(1):86–96. 10.3109/0284186X.2011.618511. 10.3109/0284186X.2011.618511 21961497

[37] Sandström M, Freedman N, Fröss-Baron K, Kahn T, Sundin A. Kidney dosimetry in 777 patients during 177Lu-DOTATATE therapy: aspects on extrapolations and measurement time points. EJNMMI Phys. Dec. 2020;7(1). 10.1186/S40658-020-00339-2. 10.1186/s40658-020-00339-2 PMC7726073 33296054

[38] Chicheportiche A, et al. Impact of single-time-point estimates of 177Lu-PRRT absorbed doses on Patient Management: validation of a trained multiple-Linear-Regression Model in 159 patients and 477 therapy cycles. J Nucl Med. 2023;64(10):1610–6. 10.2967/JNUMED.122.264923. 37500259 10.2967/JNUMED.122.264923

[39] Wang C, Peterson AB, Wong KK, Roseland ME, Schipper MJ, Dewaraja YK. Single-Time-Point Imaging for Dosimetry After [177Lu]Lu-DOTATATE: Accuracy of Existing Methods and Novel Data-Driven Models for Reducing Sensitivity to Time-Point Selection, *J Nucl Med*, vol. 64, no. 9, pp. 1463–1470, Sep. 2023, 10.2967/JNUMED.122.265338. 10.2967/jnumed.122.265338 PMC10478823 37500260

[40] Dewaraja YK et al. Aug., MIRD pamphlet No. 23: quantitative SPECT for patient-specific 3-dimensional dosimetry in internal radionuclide therapy, *J Nucl Med*, vol. 53, no. 8, pp. 1310–1325, 2012, 10.2967/JNUMED.111.100123. 10.2967/jnumed.111.100123 PMC3465844 22743252

[41] Ljungberg M, et al. MIRD Pamphlet 26: Joint EANM/MIRD guidelines for quantitative 177Lu SPECT Applied for Dosimetry of Radiopharmaceutical Therapy. J Nucl Med. Jan. 2016;57(1):151–62. 10.2967/JNUMED.115.159012. 10.2967/jnumed.115.159012 26471692

[42] Ogawa K, Harata Y, Ichihara T, Kubo A, Hashimoto S. A practical method for position-dependent Compton-scatter correction in single Photon Emission CT. IEEE Trans Med Imaging. 1991;10(3):408–12. 10.1109/42.97591. 18222843 10.1109/42.97591

[43] Götz TI, Schmidkonz C, Lang EW, Maier A, Kuwert T, Ritt P. Factors affecting accuracy of S values and determination of time-integrated activity in clinical Lu-177 dosimetry, *Ann Nucl Med*, vol. 33, no. 7, pp. 521–531, Jul. 2019, 10.1007/S12149-019-01365-6. 10.1007/s12149-019-01365-6 31119607

